# “A peculiar time in my life”: making sense of illness and recovery with gynaecological cancer

**DOI:** 10.1080/17482631.2017.1364603

**Published:** 2017-08-31

**Authors:** Eleanor Phillips, Jane Montague, Stephanie Archer

**Affiliations:** ^a^ Psychology, University of Derby, Derby, UK; ^b^ NIHR Imperial Patient Safety Translational Research Centre, Imperial College London, St Mary’s Hospital, London, UK

**Keywords:** Gynaecological cancer, focus groups, women’s health, interpretative phenomenological analysis, health experiences

## Abstract

**Purpose:** Worldwide there are nearly 1.1 million new cases of gynaecological cancer annually. In England, uterine, ovarian and cervical cancers comprize the third most common type of new cancer in women. Research with gynaecological cancer patients within 6 months of diagnosis is rare, as is data collection that is roughly contemporaneous with treatment. Our aim was to explore the experiences of women who were, at study entry, within 6 weeks of surgery or were undergoing chemotherapy or radiotherapy. **Methods:** An interpretative phenomenological analysis (IPA) of data from 16 women in five focus groups was conducted in the UK, exploring women’s experiences of being diagnosed with and treated for gynaecological cancer. **Results:** Participants conceptualized their experiences temporally, from the shock of diagnosis, through their cancer treatment, to thinking about recovery. They tried to make sense of diagnosis, even with treatment being complete. In the context of the Self-Regulation Model, these women were struggling to interpret a changing and multi-faceted illness identity, and attempting to return to pre-illness levels of health. **Conclusions:** This study adds to this under-studied time period in cancer survivorship. The results suggest that survivors’ goals may change from returning to pre-illness status to reformulating goals as survival time increases.

## Introduction

Gynaecological cancers impact the ovaries, uterus, vagina, endometrium, vulva and fallopian tubes. These cancers accounted for.3% of female cancers in 2012, with nearly 1.1 million new cases annually worldwide (Ferlay et al., 2015). Globally, cervical cancer was the fourth most frequent cancer for women and had the highest mortality rate of all female cancers, at 7.5% (Ferlay et al., 2015). In England, uterine, ovarian and cervical cancer were the third most common type of new cancer registered in women, following on from breast and lung cancers (Bannister, 2016). While gynaecological cancers make up a significant proportion of female cancers, research on survivorship is under-studied (Hughes, Whitford, Collins, & Denson, 2014; Roberts & Clarke, 2009), especially in comparison to breast cancer.

The experience of cancer involves dynamic, long-term processes, where patient needs, focus and priorities change over time (Fiszer, Dolbeault, Sultan, & Brédart, 2014; Tariman, Doorenbos, Schepp, Singhal, & Berry, 2014), especially as they move from being cancer patients to being cancer survivors (Sandsund, Pattison, Doyle, & Shaw, 2013). Cancer removal is prioritized for newly diagnosed patients, whereas those surviving long-term (at least 5 years) may begin re-evaluating life purpose and meaning (Sekse, Raaheim, Blaaka, & Gjengedal, 2010). Additionally, survivors integrate past events into accounts of the present (Roberts & Clarke, 2009), meaning that both time point and cumulative experiences are important aspects for consideration. While Hammer, Mogensen and Hall (2009) conducted their research immediately after diagnosis, most other work, both qualitative and quantitative, has taken place some time after diagnosis or treatment, from 12 months post-surgery (Roberts & Clarke, 2009), 5 years post-diagnosis (Reb, 2007; Sekse et al., 2010; Walton, Reeve, Brown, & Farquhar, 2010) and up to 16 years post-diagnosis (Molassiotis, Chan, Yam, Chan, & Lam, 2002). Whilst temporal breadth is important, research is diffused over a range of time points, with much occurring well after original diagnosis. There is little research that takes place within 6 months of diagnosis (e.g., Ekwall, Ternestedt, & Sorbe, 2003; Hammer, Hall, & Mogensen, 2013; Hammer et al., 2009). One particular gap in the time frame is that where women have completed active treatment but are not yet officially in remission from cancer. This makes this time point an area ripe for additional, explorative, qualitative research.

Previous research, both quantitative and qualitative, has investigated distress and mental health disorders in gynaecological cancer patients (Reuter, Raugust, Marschner, & Haertner, 2007; Stewart, Wong, Duff, Melancon, & Cheung, 2001), as well as support (Beesley et al., 2008; Ussher, Kirsten, Butow, & Sandoval, 2006; Walton et al., 2010), informational (Booth, Beaver, Kitchener, O’Neill, & Farrell, 2005) and psychosocial needs (Miller, Pittman, & Strong, 2003; Warren, Melrose, Brooker, & Burney, 2016). Additionally, qualitative research has expanded to explore the experience of cancer, particularly in relation to topics such as hope (Hammer et al., 2013; Reb, 2007), meaning (Akyüz, Güvenç, Üstünsöz, & Kaya, 2008; Roberts & Clarke, 2009; Sekse et al., 2010), the impact of childlessness or loss of fertility for younger women (Molassiotis et al., 2002; Roberts & Clarke, 2009) and changing family roles (Akyüz et al., 2008). Other topics identified included uncertainty and risk (Roberts & Clarke, 2009; Sekse et al., 2010), body image and sexual function (Ekwall et al., 2003; Maughan, Heyman, & Matthews, 2002; Molassiotis et al., 2002). Much of this research has been at the intersection of individual experience with healthcare providers, and often from a nursing perspective (e.g., Ekwall et al., 2003; Reb, 2007; Roberts & Clarke, 2009; Walton et al., 2010), without drawing on relevant psychological theory.

One theory that has appeared in gynaecological cancer research is the self-regulation model (SRM) (Leventhal et al., 1997). The SRM is often described as a common sense model of illness experience and combines multi-dimensional illness representations, goals formulated in response to the health threat, and coping strategies (Leventhal, Leventhal, & Contrada, 1998). It is a conceptualisation of a health problem that models a cyclical process through goal setting, coping strategy selection and implementation, and then evaluation and readjustment (Boekaerts, Maes, & Karoly, 2005). The SRM is a good fit with qualitative research into health experiences because it is essentially phenomenological (Leventhal et al., 1998), thus meshing well with qualitative approaches such as interpretative phenomenological analysis (IPA) (Smith, 1996). IPA and the SRM have been combined to study varioushealth issues including infertility (Phillips, Elander, & Montague, 2014), early stage dementia (Harman & Clare, 2006), non-epileptic seizures (Green, Payne, & Barnitt, 2004) and vitiligo (Thompson, Kent, & Smith, 2002). Bruner and Boyd (1999) used the SRM as a conceptual framework for developing a sexual functioning measure. Bradley, Calvert, Pitts and Redman (2001) applied it to their research with gynaecological cancer patients, focusing on illness identity linked with the circumstances of diagnosis and its impact on future evaluations of cancer recurrence. The elements of the SRM are valuable concepts in interpreting experiences and their impact, and in combination with IPA as a phenomenological approach.

Most qualitative gynaecological cancer research has used interviews, which give a particular perspective. Turning to other forms of data collection, such as focus groups (FGs) (Ussher et al., 2006), may illuminate different aspects of experience, particularly adding to our understanding of how social contexts might influence illness representations. The aim of the present study was to explore the experiences of women who had received treatment for gynaecological cancer, but are not yet officially in remission.

## Methods

Five FGs explored the experience of living with gynaecological cancer. The data were analysed using IPA, which investigates how people make sense of health experiences. As an experiential model of health and illness, the SRM is a good fit with phenomenological approaches such as IPA (Smith, Flowers, & Larkin, 2009). While IPA has been traditionally problematic for analysing FG data, recent methodological suggestions provide a basis for carrying out this kind of analysis (Palmer, Larkin, de Visser, & Fadden, 2010; Tomkins & Eatough, 2010). Taking account of difficulties, and building on work cited above, we developed our own analysis approach, which is described later in this article (Phillips, Montague, & Archer, 2016).

## Participants

Sixteen women (age range 31–79 years; mean age 60) who had participated in a yoga intervention took part in these FGs. The participants were a subset of a larger study of women being treated for gynaecological cancer recruited from a large hospital in the UK. At the start of the study, participants were either within 6 weeks of surgery or were receiving chemotherapy or radiotherapy for cancer of the ovary, cervix, vulva, uterus, fallopian tube or peritoneum. Most still had to undergo their first scan post-treatment but at the focus group the majority had completed treatment. They all felt well enough to participate in a programme of gentle yoga.

## Data collection

Participants were invited to attend one of a series of FG discussions facilitated by SA, and some with a yoga instructor also participating (see Table 1). Focus groups were conducted between June 2011 and June 2012. The participants requested the instructors’ presence to allow them to give direct feedback about programme-related components. The instructors only participated in the discussion when directly addressed. The FGs were guided by a range of open-ended questions, such as the type of cancer, the treatment they had, and their experiences around health and illness, specifically about cancer. Each FG was audio recorded and transcribed verbatim. All names used here are pseudonyms. The study conformed to the British Psychological Society’s ethical standards (British Psychological Society, 2009), and participants were fully briefed on the purpose of the discussion. The overall research project received NHS Research Ethics Committee approval and local Research & Development approval.

**Table 1.** Focus-group participants.

	Participant name	Age	Cancer	Stage	Treatment
Focus group 1	Liz	55	Ovarian	Stage 3	Surgery/chemo
	Kathleen	68	Primary Peritoneal	Stage 3	Chemo
	Lucy	32	Cervical	Stage 2	Surgery/radio
	Alice	57	Ovarian	Stage 3	Surgery/chemo
	Sheila	63	Endometrial	Stage 3	Surgery/radio
Focus group 2	Helen	61	Endometrial	Stage 1	Surgery
	Brenda	79	Endometrial	Stage 1	Surgery/Radio
	Lily	77	Endometrial	Stage 1	Surgery
Focus group 3	Brenda	79	Endometrial	Stage 1	Surgery/radio
	Sharon	63	Ovarian	Stage 4	Surgery/chemo
	Rose	56	Endometrial	Stage 1	Surgery
Focus group 4	Moira	52	Ovarian	Stage 1	Surgery/chemo
	Jane	69	Endometrial	Stage 1	Surgery/radio
	Betty	65	Endometrial	Stage 1	Surgery
Focus group 5	Dorothy	56	Endometrial	Stage 1	Surgery
	Sheena	64	Ovarian	Stage 1	Surgery/chemo
	Judith	57	Endometrial	Stage 1	Surgery

## Analysis approach

Initially, transcripts were coded using a standard IPA approach, noting how and where participants made sense of their own experiences (Smith et al., 2009). All aspects of the data were coded, spanning yoga, cancer and treatment. One set of themes, discussing the yoga programme, was presented in a previous paper (Archer, Phillips, Montague, Bali, & Sowter, 2015). We then turned our attention to interactions and group concepts. Each transcript was coded again, identifying the ways in which participants made sense of their world and their interactions with others, as well as coding conversations between participants, where they made meaning together.

During this process, we developed a model where the individuals’ interpretations of their own experiences lay at the centre, wrapped around by layers of widening social contexts. We looked at how participants related their accounts to those of other patients, other group members, their social circle and the wider world. These layers were used to interpret participants’ experiences in the analysis presented here, concentrating on themes that were prevalent among, and representative of (Smith, 2011), the participating individuals.

## Analysis

The women’s accounts of health and illness fell into a temporal arc with three parts: (1) the shock of diagnosis (past), (2) having treatment to remove the cancer (mostly past) and (3) hoping for recovery (present and future). These three themes encompass both significant aspects of the women’s experience of cancer and general ways of storytelling about their lives.

## “Like being mowed down by a bus”: The shock of diagnosis

In the early parts of their accounts, participants described their diagnoses. Within these descriptions they attempted to make sense of the suddenness and seriousness of what happened, which often occurred during regular check-ups:

I just went for a routine smear test in September and they found I’d got a tumour. And I went to [name of hospital] and they took a biopsy, and they told me that it was cancerous, so then I got referred to [name of hospital] where within 3 weeks I’d had a full hysterectomy [laugh] oh this was—I had to wait ‘til—I found out on Christmas eve, actually, about it, that it was cancerous and I’d got to wait over Christmas. (Lucy, FG1)

Lucy’s account focused on practicalities—times and places—and minimized the emotional impact of diagnosis while including dramatic contrasts: between a “just…routine” test and the serious diagnosis; the speed of some events, against a wait over Christmas. Her laughter further reinforced these incongruities. She had little time to become accustomed to the idea of cancer before it was removed. Others reflected on the shocking nature of their diagnosis:

I mean it’s such a traumatic experience, isn’t it? Because you have to go through the day when they say to you you’ve got cancer, and when you don’t think you have, it didn’t even enter my head, the shock was [pause] unbelievable, awful, and then you’ve got to go through the MRI scan, you know, the things like that, and all of that is traumatic, isn’t it? (Lily, FG 2)

Where the time of year was significant for Lucy, Lily’s description of “the day…you’ve got cancer” gave a precise time-point where cancer started for her. She described the shock and trauma associated with diagnosis, which the pause emphasized, as did other participants (for example Alice (FG1) mentioned “incredible lows” and “shocks”). Lily’s account switched between “we”, “you” and “I”, which, along with the tag question (Lakoff, 2004) “isn’t it” that peppered her account, oriented her experience to that of the other women present. She attributed these reactions to all, assuming them as common across the group. Accounts of diagnosis, therefore, showed a contrast between conveying neutral facts, possibly even a dramatic, exciting story, and clearly apparent trauma and devastation.

For many participants, cancer had few identifiable symptoms, and left them trying to interpret their serious diagnosis against a background of having not felt ill:

Moira: I didn’t have a definite diagnosis until after the operation. I’d just gon-, I’d gone to the GP for something else because I was well having pains here and he just looked at me and said there’s a lump there [laughs] I had no idea. How can you = 

Jane: = I had no pain anywhere = 

Moira: = how can you not notice something like that [laughs]?

Jane: And I didn’t lose weight or anything like that and so you know there were no pointers to say there was anything wrong, until a very slight bleed. (FG 4)

Moira and Jane co-constructed an account, suggesting a similar experience and showing a dissonance that many participants also struggled to understand between their sense of embodiment and the cancer diagnosis. Underlying this were ideas about what it “should” mean to have cancer: that it was expected to be associated with symptoms like weight loss, pain and feeling ill. Their experience did not fit with their expectations of what cancer should be. These ideas were reinforced by others’ opinions on what they expected to see in those with cancer:

Brenda: And when you meet people out and they say have you really, you don’t look as though you’ve been, and they say you don’t look as though you’d been ill, but I’ve never felt ill.

Rose: Yeah, that’s the same as me I’ve never felt ill at all, really.

Sharon: Well, I felt very very ill when I first started, didn’t suspect anything like cancer, I must admit, but I was really really ill and probably didn’t realize quite how ill I was. (FG 3)

Not only did participants not necessarily feel ill, but, as Brenda highlighted, they did not always appear so to others. Some did feel unwell before diagnosis, however, and Sharon’s differing experience emerged during this exchange. In contrast to Brenda and Rose’s stories, Sharon emphasized how “very” and “really” ill she was. Even though she felt differently, however, this was still not associated with cancer. Being bound together by their illness and highlighting commonalities where they existed (such as none of them suspecting cancer) indicates a level of security with one another in recounting their experiences.

The cancer experience started suddenly for most participants, with diagnosis being a surprise, and few symptoms to indicate the possibility of something serious. The women tried to make sense of what cancer meant for them, especially the lack of symptoms and not feeling (or looking, according to others’ reports) ill at all. There was disruption to routine, particularly when their diagnosis occurred during a medical consultation for a seemingly mundane issue. The women demonstrated two distinctive ways of talking about their disease, as either a factual account, or one that foregrounded the shock and horror of cancer. Following diagnosis, the women discussed their next significant time point as that of treatment.

## “You’ve taken it away, I haven’t got it now!”: What is treatment?

Treatment was discussed less frequently within the groups than diagnosis or recovery, perhaps because the women had been in contact during treatment while participating in the yoga programme, and were therefore more familiar with each other’s experience at that time. Participants made some unexpected interpretations of what they considered treatment to be, however:

Betty: I’m Betty. I’ve had ovarian cancer and I’ve not had any treatment, have I?

Janine (Betty’s daughter): You’ve had a full hysterectomy.

Betty: Sorry?

Janine: Full hysterectomy. (FG 4)

Betty sought clarification about her treatment and her daughter, Janine, located her hysterectomy within this category. It was unclear how Betty interpreted her surgery: perhaps because as hysterectomy occurs among this age group for other conditions, she might not have perceived it as specific to her cancer. For others, not having had surgery required explanation:

So it hadn’t actually affected any of the organs as such, but there was no operation I could have. I had a biopsy by through having laparoscopy and all this so called seeding, which is tiny little cancerous cells inside the momentum [sic], the only way to cure it really was by purely chemo, so I’ve had 6 doses of the chemo, which was exactly the same treatment as you have for ovarian cancer, the taxol and the carbonplatin with all the side effects [laughs]. (Kathleen, FG 1)

The phrase “no operation I could have” emphasized surgery as not available for Kathleen, rather than not needed. This suggests that requiring surgery may give validity to her diagnosis, and normalize her experience to other participants, whose treatment programme was surgery *then* chemotherapy and/or radiotherapy. Kathleen highlighted receiving “purely chemo”, using drugs’ names without explanation and noting similarities in treatment protocols and side-effects. This emphasized experience shared with the group and established her group membership.

In contrast with the lack of symptoms prior to diagnosis, participants expected and found that treatment would cause patients to “feel ill” (Lily, FG 2) and generally treatment caused more physical effects than existed prior to diagnosis:

Well, from a radiotherapy point of view it was, it was severe fatigue but it wasn’t, you did want to go to sleep but it was such a heavy eyed feeling that everywhere you were, either on your feet or sitting down, you just wanted to shut your eyes, you know, and it was such a fatigue type, a draining feeling. And the only other side-effect I had was nausea, but they did give me some tablets, which helped, but it never actually went away and it was there all the while. (Sheila, FG1)

Sheila minimized her side-effects from radiotherapy, highlighting severe fatigue and nausea as being the only ones she experienced. These affected her differently: she was weighed down by the exhaustion associated with radiotherapy, emphasized by phrases like feeling “heavy eyed” and it being “draining”. In comparison, the nausea she experienced appeared less bothersome and was somewhat treatable. Unlike most participants, Sharon had emphasized feeling extremely ill before diagnosis (see quote in previous section), and treatment improved her well-being in comparison:

But once I started having the treatment then I got better and better and you know, I feel I’ve done really well through the treatment and operation and everything, compared to how, well about this sort of time last year when it first started. (Sharon, FG 3)

Sharon felt she had underestimated her illness and indicated surprize at how much better she felt once treatment started. In comparison, most other participants had not felt ill, and most of their discomfort with cancer arose during treatment and due to those side-effects. Health at diagnosis was a key comparison for evaluating the side effects of treatment, and later recovery.

The women discussed treatment less than diagnosis or recovery, perhaps because it was something they were not in control of; they were following a protocol they were given to achieve their goal of having the cancer removed. While diagnosis was still significant to the participants, treatment appeared less so. Surgery was not necessarily considered treatment, although the women tried to highlight the aspects they had shared. While many found cancer itself largely symptom free, treatment caused many side-effects. The significance of side-effects, and the perception of what treatment entailed, were important elements in how participants made sense of it. These also affected the trajectory of the women’s recovery.

## “Touch wood, hopefully, everything will be alright”: Defining recovery

Most participants had finished treatment whilst participating in the yoga intervention and their accounts were focused on recovery. Many described having had cancer but having been cured, particularly during introductions, with factual statements such as “I had endometrial cancer” (Sheila, FG 1) and “I’ve had ovarian cancer, which was removed” (Moira, FG4). The latter, in particular, positioned cancer as over, but defining recovery was not always as straightforward:

As far as I’m concerned it’s gone, the surgeon says it’s gone, so I hope that that is right. (Jane, FG 4)

Jane cited medical opinion and authority to define her recovery and surgery as an important marker for treatment. Despite stating her surgeon’s assessment of her cancer she demonstrated a glimmer of doubt, suggesting that shedding the label of cancer required further confirmation. For others, recovery meant getting back to normal:

Rose: I think what surprised me is, at first I sort of really well, everybody couldn’t believe how well I was doing, but I wasn’t doing anything I was resting, walking = 

Brenda: = Yes, that’s what I found = 

Rose: = You know, sort of like, so I’d hardly got any pain or anything but then it’s when a few months after, when you start doing stuff you think well should it be hurting now? You know, and I think it’s the fact that, well the doctor said it sort of takes up to a year to get back to normal. (FG3)

Recovery occurred in stages, starting with feeling well and not feeling pain, basic mobility and then resuming pre-cancer roles and activities, essentially returning to “normal”, which was the goal of participants. As Rose described above, the expected timeline for recovery was not clear. They gleaned information from their doctors, observations of others, pain experience and reactions to medication, among other things. As with the cancer label, participants viewed their recovery through the eyes of family and friends, which was not always helpful, as others’ perceptions did not necessarily mesh with participants’ views of their own capabilities and situation:

Your friends and your family are, you know, the sort of, the sympathy bucket is only so big, isn’t it? And after you’ve, you know, you’ve physically seem to have recovered and you can do pretty much all the things that you always did before, people forget, which is absolutely right, healthy and proper. They forget that you’ve been very poorly or potentially even may be poorly again. (Judith, FG5)

Participants may be seen by others as being fully recovered when they are not; similarly, others may remain unaware of future challenges, again, causing a clash of perceptions. For Judith, support from others was tied to receiving “sympathy”, whereas from the other participants, support entailed sharing common experiences. Physical concerns for the future related to getting back to previous activity, and emotional dimensions centred on the uncertainty and worry associated with future tests that others described:

And I think there’s a *fear*. I think there’s a great fear that I’m sure we’ve all experienced that [yeah]. A fear of the unknown, really, because, yes we are in a fortunate position, we go for scans; but as the appointment comes for my next scan, which is coming very very soon, I know, I’m really hoping that, you know, I could put my yoga experience things with the breathing and the relaxation into practice because I know I will be very worried. (Sheila, FG 1)

Appreciation of the available monitoring was balanced by the anxiety it generated. As in the diagnosis stage, worry and anxiety seemed easily attributed to the whole group. Sheila generalized this fear to other participants, contrasting with an earlier, personalized statement in the discussion that she “felt very good, to be honest”. Including “to be honest” indicated caution about statements of recovery (Edwards & Fasulo, 2006), being sensitive to others recovering more poorly, or not at that stage in treatment.

Recovery was, perhaps not unexpectedly, an area of key importance to participants. Defining recovery was difficult; it may have included receiving an official statement of remission from the medical team, appearing well to friends and family and regaining old roles and engaging in old activities. In addition, there was no clear point at which recovery had (at least for these women) been achieved, and good health, even from a medical standpoint, was conditional and could be lost with future scans. This uncertainty stood in contrast to the suddenness and definiteness of diagnosis. The cycle of test and answer led to worry and stress, and reliance on hope for the future, because even clear statements from doctors or scans appeared somewhat equivocal. The participants were aware of the range of others’ experiences and framed their accounts of their own recovery to be considerate to these.

## Discussion

The women’s experiences of gynaecological cancer described a temporal arc from recollections of the shock of diagnosis, through having their (mostly complete) treatment to remove the cancer and projecting hope for recovery into the future. There were fewer accounts of treatment than of diagnosis and recovery, and treatment seemed less troubling to the women, with their accounts focusing largely on physical side-effects. Other research has suggested that the main need of cancer patients at diagnosis is to have the cancer removed (Hammer et al., 2009), with surgery the only treatment deemed curative by patients (e.g., Ekwall et al., 2003). As these women had mostly finished treatment, this stage was, perhaps, of less relevance to them; as many said, the cancer had been removed. This might also reflect the self-selection of participants as able to take part in the yoga programme during treatment, so while this experience is likely not representative of all gynaecological cancer patients, it shows that the range of experiences can be large and some may feel stronger and more physically capable, despite the effects of treatment.

Many issues that our participants struggled with fit the illness identity (label and symptoms of illness) portion of the self-regulation model (Leventhal et al., 1998), echoing findings from Bradley et al. (2001). Participants emphasized the shock of diagnosis (Akyüz et al., 2008; Leal et al., 2015), particularly when they had experienced few symptoms, as is common with gynaecological cancers, which are often asymptomatic or attributed to other issues such as irritable bowel syndrome or ageing (Goff, Mandel, Melancon, & Muntz, 2004). There is a perception that cancer “should” be signalled by serious symptoms (Bradley et al., 2001), as the women here described, meaning there is a contradiction between that label and the lack of symptoms; this enriches our understanding of gynaecological cancer representations within the context of the SRM (see Leventhal et al., 1998). Bradley et al. (2001) linked their participants’ illness identity to the symptoms they experienced at diagnosis, projecting this forward to explain anxiety about future recurrence. Our women struggled with this issue, too, and also tried to reconcile input from various sources that went to create that label. For example, a doctor may have told them that the cancer was removed, but they were still awaiting confirmatory scans. Their own sense of embodiment could not identify cancer, and others’ perceptions of their illness (or lack of it) were another source of uncertainty. The variety of sources providing observations contribute to complexity of the “cancer” label as an illness representation in the SRM.

The SRM includes concepts of time within illness representations (Maes & Karoly, 2005). While diagnosis was a clear and memorable time point, the women noted the uncertain timeline of their recovery; another part of illness representations in the self-regulation model (Leventhal et al., 1998). The women mentioned unexpected delays in returning to pre-illness levels of mobility and function and difficulty knowing when these would return. Comparisons with other research where participants were later on in survivorship suggests that recovery goals may change over time from a desire to return to pre-illness function to trying to find a “new normal” (Molassiotis et al., 2002; Reb, 2007; Sandsund et al., 2013). This potential for changing goals in the time period after treatment could benefit from further investigation. In addition, this suggests a disconnect between expectations and actual recovery, which could benefit from better understanding and communication to patients. Some women in our study mentioned that information from participants “further on” in the recovery process was helpful. This kind of informational/experiential support from other patients has been found to be helpful in other areas of women’s health, such as infertility (Malik & Coulson, 2008; Phillips, Elander, & Montague, 2014). This illustrates the value of using IPA to develop insights into the application of the SRM with particular conditions.

One significant difference between this study and much other cancer research is that data collection was roughly contemporaneous with treatment. For the women in this study, these were recent experiences, whereas many studies took place much later, from 12 months post-surgery (Roberts & Clarke, 2009) to up to 16 years post-diagnosis (Molassiotis et al., 2002), in which time, narratives will almost certainly become reconstructed as they are told and retold (Smith, 1994). Additionally, the potential for changing health goals over time, with a “new normal” being created, suggests that better understanding is needed of these developments, particularly as this changing need may require support from healthcare professionals and those in support roles. Additional research is required to investigate this in order to better address changing patient needs over time.

There are aspects from previous gynaecological cancer research that did not emerge here. This can perhaps be attributed to the effect of being in a group, or because the group was in the context of a yoga programme, within which context some topics were not deemed appropriate. The women did not discuss the possibility of death, for example, and there was no discussion of marital difficulties or sexual problems, which have occurred in other work (Reb, 2007; Roberts & Clarke, 2009). Sekse et al. (2010) point out that topics like death and sexuality are difficult to discuss, even in a group of survivors, although they found a similar unspoken understanding within their participant groups to that which emerged here. The shared experiences reported here reflect what Ussher et al. (2006) found: that support groups enable community, acceptance and sharing of information. Their reflections on the experience of all these stages of their diagnosis, treatment and recovery demonstrate their importance to these participants, whatever the context. Healthcare professionals and other support staff should be aware of the range of patients’ experiences, and that not all individuals may experience the same concerns or trajectory post-treatment. Professionals should be sensitive to patients’ (potential lack of) willingness to discuss particular topics within groups and provide appropriate environments to do so.

This research explored participants’ experiences of gynaecological cancer shortly after treatment. There is little research with cancer patients in this time period, and no work with this group conducted in the past 5 years that we were able to identify. Comparing this study with other research findings suggests that post-cancer experiences may evolve with time, and survivors may experience a change from attempting to regain their pre-diagnosis norm to attempting to find a new goal and equilibrium. The themes showed experience as a temporal arc that began with a shocking diagnosis, but extended for an uncertain amount of time into the future. Many of the issues addressed fell into the concept of illness identity from the SRM, showing participants struggling to make sense of their cancer label. Contradictory messages from different members of their medical team, their own embodied sense and observations of others obscured where the illness could and should begin and end. The SRM provides a valuable model and further work on other aspects of the SRM, such as timeline and changing goals, could be fruitful.
